# A strategic framework for extending the Egyptian geodetic CORS network in support of vision 2030

**DOI:** 10.1038/s41598-026-67304-4

**Published:** 2026-08-25

**Authors:** Mohamed El-Tokhey, Mohamed Mamdouh, Tarek Hassan

**Affiliations:** Faculty of Engineering, Ain Shams University, Cairo, Egypt

**Keywords:** GNSS Networks, CORS, Egypt’s vision 2030, Network extension, Environmental sciences, Ocean sciences

## Abstract

The national development strategy, Egypt’s Vision 2030, is catalyzing large-scale expansion in desert regions through infrastructure networks, agricultural reclamation, and urban communities. Mega-projects such as the New Delta, the New Valley, and developments along the North Coast and the Red Sea Coast necessitate a precise and unified geodetic reference network for Global Navigation Satellite Systems (GNSS). However, the current configuration of the national Continuous Operating Reference Stations (CORS) network remains concentrated in the Nile Valley and Delta regions, leaving these newly developed mega-projects insufficiently covered. This study proposes strategic extensions to the Egyptian CORS network to ensure geodetic consistency and support the planned large-scale developments in the coming years. The proposed framework in this study integrates GNSS network design principles with mathematical modeling and rigorous adjustment techniques to estimate the geodetic accuracies of the proposed stations and baselines. The framework was developed based on the existing Egyptian CORS network, which comprises 49 stations. To extend coverage to the targeted zones, 66 new stations are proposed, expanding the CORS network to a total of 115 stations. For the accuracy assessment, GNSS observations from the existing 49 stations were collected and processed to derive the coefficients required for baseline accuracy estimation. The results demonstrate the consistency and robustness of the proposed CORS extensions, with positional standard errors below 1.8 cm, confirming their reliability for regional geodetic infrastructure and centimeter-level positioning applications in support of Egypt’s Vision 2030.

## Introduction

Egypt’s Vision 2030 outlines several key initiatives across the agricultural, industrial, and urban sectors. These initiatives focus mainly on reclaiming and expanding agricultural land in desert regions while improving and modernizing irrigation systems. These development targets are projected to continue through 2035. Furthermore, Egypt’s urban development plan outlines new cities and urban clusters extending beyond the Nile Valley and Delta zones to reduce pressure on existing settlements.

By 2030, Egypt aims to increase its cultivated land from approximately 9.7 million to over 15 million acres. New Delta is one of the mega-projects aiming at reclaiming desert land (approximately 2.2 million acres) for agriculture in the Western Desert, west of the Nile Delta. The project routes a large artificial river west of the Nile, supplying reclaimed desert with water and creating new communities^10^. In addition, near the New Delta project lies the El-Maghara project, which is located in the northeastern part of the Qattara Depression. This project aims to reclaim about 400,000 acres based on groundwater basin resources^8^.

Other initiatives in Egypt’s desert-reclamation program include the Toshka and New Valley projects. In these projects, water is channeled from Lake Nasser into the southwestern desert to create new irrigated agricultural lands, lakes, and settlement areas^4^. Moreover, the development of the New Valley governorate includes large-scale solar and infrastructure projects essential for supporting population growth and agro-industry and for managing environmental impacts in fragile oasis systems^25^.

Another region targeted for large-scale development is the western zone of the North Coast. This includes a high-capacity, multi-lane highway of approximately 280 km, extending from El-Alamein to Sallum and passing through Marsa Matruh. Additionally, the development program encompasses the establishment of the Sallum and Gargoub ports^23^. Besides, the initiative aims to reclaim desert land in the area by utilizing an integrated water strategy that combines groundwater extraction with seawater desalination^9^. Similarly, major development efforts are underway along the Red Sea Coast, where significant mega-projects are planned. This includes the additional upgrades to Sokhna and Safaga ports, alongside the establishment of the Golden Triangle economic zone as an integrated hub for mining, industry, agriculture, and tourism.

As Egypt continues to implement the planned large-scale development projects, the need for uniform and precise geodetic control becomes increasingly critical. Today, Global Navigation Satellite Systems (GNSS) positioning has become the backbone for establishing such precise geodetic networks, ensuring nationwide consistency and long-term positional stability when the various error sources are properly modeled and controlled^15^. To operationalize this reference framework in practice, Continuous Operating Reference Stations (CORS) networks are capable of delivering precise real-time and post-processing positioning services^18^. These networks represent the foundation for accurate surveying, mapping, and construction purposes through static, Real-Time Kinematic (RTK), or Virtual Reference Station (VRS) positioning techniques^28^.

Despite recent development initiatives in Egypt, the existing CORS network remains concentrated mainly in the Nile Delta and Nile Valley regions, leaving the new development areas uncovered. Therefore, extending the CORS network coverage is essential to support nationwide geospatial integration and ensure geodetic accuracy across the ongoing and future development projects. This expansion is also crucial for improving inter-agency coordination and supporting modern digital and smart city systems.

This research aims to propose strategic extensions to the Egyptian CORS network, in alignment with the country’s national development vision. The targeted regions include the New Delta, the western zone of the North Coast, Toshka, the New Valley, and the Red Sea Coast. The objective is to provide a framework for establishing coherent extensions to enhance surveying accuracy and support large-scale infrastructure implementation. In addition, the study estimates the expected positioning accuracies of the proposed extensions through rigorous GNSS data processing and network analysis based on the existing CORS configuration.

The following parts of this paper are organized as follows. In “The Egyptian HARN and CORS networks with the proposed extensions” section, the current configurations of the GNSS networks in Egypt are presented, along with the proposed extensions. Then, “Estimated accuracy of the GNSS baselines and stations” section discusses the mathematical basis for estimating the expected accuracies of the new baselines and stations. Later, in “The estimated positional standard errors of the proposed CORS stations” section, the obtained results for the five proposed extensions are presented. Finally, the conclusions of this research are given in “Conclusions” section.

## The Egyptian HARN and CORS networks with the proposed extensions

The Egyptian High Accuracy Reference Network (HARN) is a geodetic network established by the Egyptian Survey Authority (ESA) in 1995 to provide high-accuracy geodetic control for surveying and mapping applications. It comprises 30 stations covering the entire territory of Egypt, with an average spacing of approximately 200 km between stations, achieving an accuracy of 1:10,000,000. The HARN network was connected to the global network of International GNSS Service (IGS) stations, aligning it to the International Terrestrial Reference Frame 1994 (ITRF1994)^5^.

**Fig. 1:**
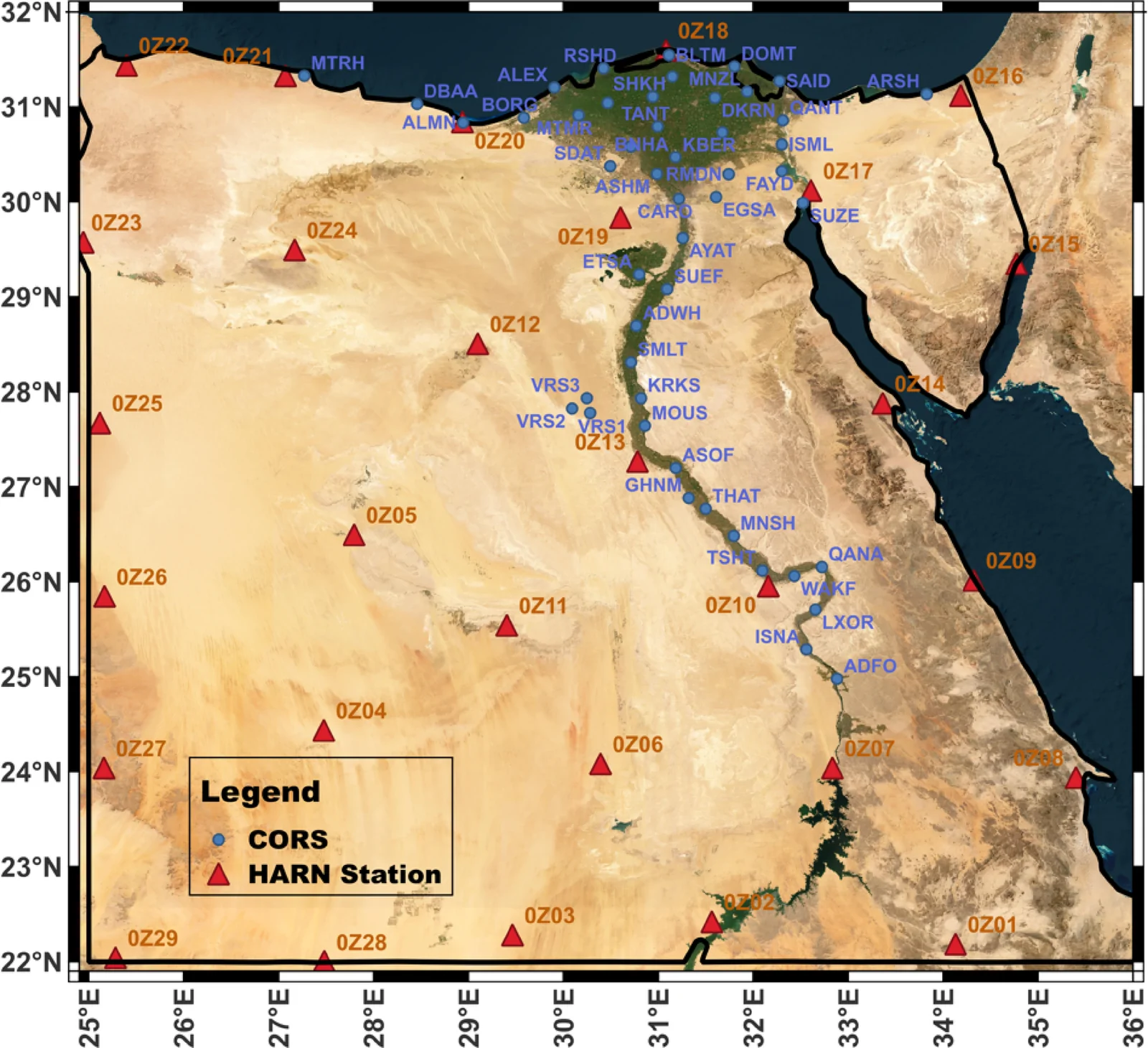
Station distribution of the Egyptian HARN and CORS networks. *Generated using QGIS version 3.42.2 *(https://qgis.org).

In 2011, the Egyptian CORS network was established, initially consisting of 40 continuously operating stations. The network was connected to the IGS network and adopted ITRF2008 as its static datum with 2011.811 as the reference epoch. More recently, ESA extended the network by adding nine stations, increasing the total number to 49. In 2025, the entire network was readjusted and connected to ITRF2020^3^. The complete procedural details and outcomes of this adjustment are documented in^20^. Figure 1 shows the current configuration of the Egyptian HARN and CORS networks.

**Fig. 2:**
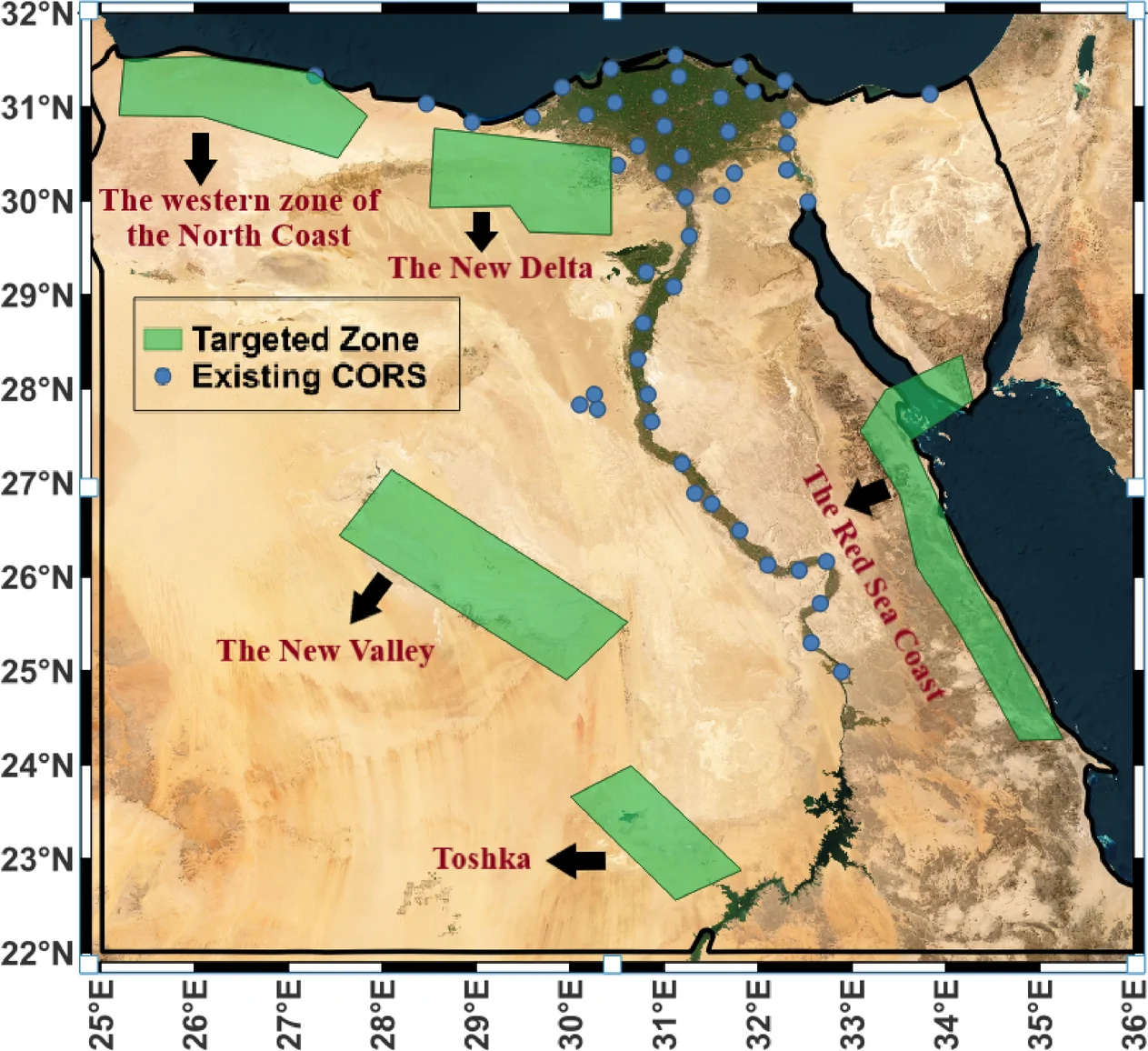
The targeted regions for development according to Egypt’s Vision 2030. *Generated using QGIS version 3.42.2 *(https://qgis.org).

Unlike the nationwide distribution of the HARN stations, the CORS network remains largely concentrated around the Nile Valley, the Delta, and the North Coast regions. As described in the introduction section, this paper aims to propose the necessary extensions that can serve the planned national mega projects in the New Delta, the western zone of the North Coast, Toshka, the New Valley, and the Red Sea Coast. Figure 2 depicts the regions targeted for development with respect to the current configuration of the CORS network.

For the targeted extensions, the locations of the proposed stations are chosen so that the baseline configurations are triangular and quadrilateral, with spacings of 15–95 km between stations. Most of the longer baselines in the proposed extensions are to connect the new stations to the Egyptian network, relying on the nearest CORS or HARN stations. Therefore, the selected stations are distributed to minimize coverage gaps and provide a well-conditioned network geometry for subsequent adjustment.

In addition to the geodetic design criteria, practical site selection considerations were also taken into account. These included the availability of reliable power and telecommunications to support continuous operation and data transmission, accessibility for routine maintenance, physical security, and the potential for future network expansion. Whenever possible, suitable government-owned facilities were preferred to facilitate installation, operation, long-term maintenance, and cost-effective implementation.

Another important factor in site selection is the geological stability of the location. Selecting locations with minimal susceptibility to seasonal ground deformation is preferred for establishing CORS to ensure reliable long-term performance^1,7,21^. All of these considerations align with internationally recognized recommendations for CORS network implementation^6^.

**Fig. 3:**
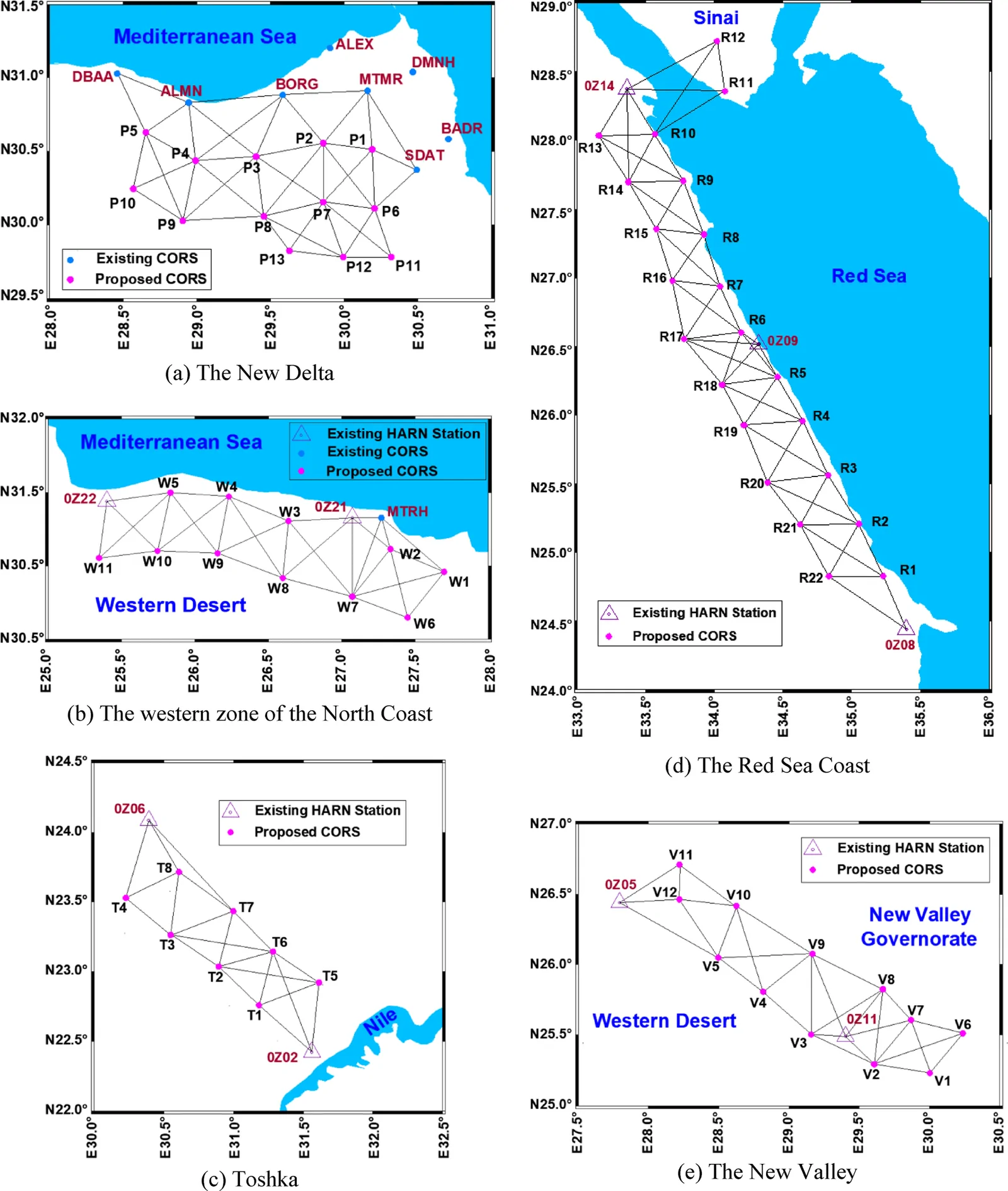
The proposed extensions of the Egyptian CORS network. *Generated using QGIS version 3.42.2* (https://qgis.org).

The proposed extensions in the five zones (i.e., the New Delta, the western zone of the North Coast, Toshka, the New Valley, and the Red Sea Coast) are presented in Fig. 3. In addition, Table 1 summarizes the number of proposed CORS, existing CORS, and HARN stations included in the proposed extension of each developing zone.

**Table 1:** The number of proposed CORS, existing CORS, and HARN stations included in the proposed extension of each developing zone.

Developing zone	No. of proposed CORS	No. of existing CORS	No. of HARN stations
The New Delta	13	5	0
The western zone of the North Coast	11	1	2
Toshka	8	0	2
The Red Sea Coast	22	0	3
The New Valley	12	0	2

For the New Delta zone, it is proposed to establish 13 CORS; nine to serve the New Delta project and four for El-Maghara project. Five existing CORS (SDAT, MTMR, BORG, ALMN, and DBAA) are used as reference stations to connect the new stations to the Egyptian network, see Fig. 3a. In another zone, the western zone of the North Coast, 11 CORS are proposed, as shown in Fig. 3b. For connection with the national network, one existing CORS (MTRH) and two HARN stations (OZ21 and OZ22) are included with the proposed extensions.

The proposed CORS network extension in Toshka includes eight new stations (T1–T8). These stations are to be connected to the Egyptian network through two HARN stations (OZ02 and OZ06), see Fig. 3c. For the Red Sea Coast, 22 CORS stations are proposed, stretching to serve about 500 km along the coast. Stations R11 and R12 are also included to serve the South Sinai area. For this proposed extension, three HARN stations (OZ08, OZ09, and OZ14) are used for connection with the national network, as shown in Fig. 3d.

The last proposed extension serves the New Valley zone, where 12 CORS are proposed. As no CORS exist in this region, the proposed stations are to be connected to the national network through two HARN stations (OZ05 and OZ11), as shown in Fig. 3e. Table 2 presents an overview of the included baselines in the proposed extensions.

**Table 2:** The number of baselines included in the proposed extension of each developing zone, along with statistics of the baseline lengths.

Developing zone	No. of baselines	Baseline lengths (km)		
		Min.	Max.	Average
The New Delta	45	31	71	45
The western zone of the North Coast	32	24	71	50
Toshka	22	42	95	55
The Red Sea Coast	62	16	88	49
The New Valley	31	27	82	55

## Estimated accuracy of the GNSS baselines and stations

Different approaches can be used to estimate the accuracy of GNSS baselines in any proposed network. For instance, the variance of a GNSS baseline can be estimated using an empirical distance-dependent model^18^. In another approach, an empirical form can be used to relate the standard error of a proposed GNSS baseline to its observation time and length^13^. In this study, the approach presented in^27^ is employed, and so, it will be reviewed in the following parts of this section.

The standard error of a GNSS baseline ($\:{\sigma\:}_{b}$) can be expressed in terms of the observation duration (* T*), the baseline length ($\:{l}_{b}$), the number of observed satellites (* SV*), and the Positional Dilution of Precision (* PDOP*) as^27^:

1 $$\:{\sigma\:}_{b}=\:{\alpha\:}_{o}+{\alpha\:}_{1}\:{l}_{b}+{\alpha\:}_{2}\:ln\left(T\right)+\:{\alpha\:}_{3}\:SV+\:{\alpha\:}_{4}\:PDOP$$

where $\:{\alpha\:}_{o}$, $\:{\alpha\:}_{1}$, $\:{\alpha\:}_{2}$, $\:{\alpha\:}_{3}$, and $\:{\alpha\:}_{4}$ are coefficients that can be determined using the Least Squares (LS) adjustment in its parametric form. This form is expressed mathematically as $\:L=AX+{\upepsilon\:}$, where * L* denotes the observations vector, and $\:{\upepsilon\:}$ is the noise. The coefficient matrix (* A*) can be expressed for * n* observations as:

2 $$ A = ~\left[ {\begin{array}{*{20}c} 1 & {l_{{b1}} } & {\ln ~T_{1} } & {SV_{1} } & {PDOP_{1} } \\ 1 & {l_{{b2}} } & {\ln ~T_{2} } & {SV_{2} } & {PDOP_{2} } \\ \vdots & \vdots & \vdots & \vdots & \vdots \\ 1 & {l_{{bn}} } & {\ln ~T_{n} } & {SV_{n} } & {PDOP_{n} } \\ \end{array} } \right] $$

Considering equal-weight observations, the vector of unknowns $$\:X={\left[\begin{array}{ccccc}{\alpha\:}_{o}&\:{\alpha\:}_{1}&\:{\alpha\:}_{2}&\:{\alpha\:}_{3}&\:{\alpha\:}_{4}\end{array}\right]}^{T}$$ can be determined using the LS solution^12,30^:

3 $$\:X={\left({A}^{T}A\right)}^{-1}\:{A}^{T}L$$

**Fig. 4:**
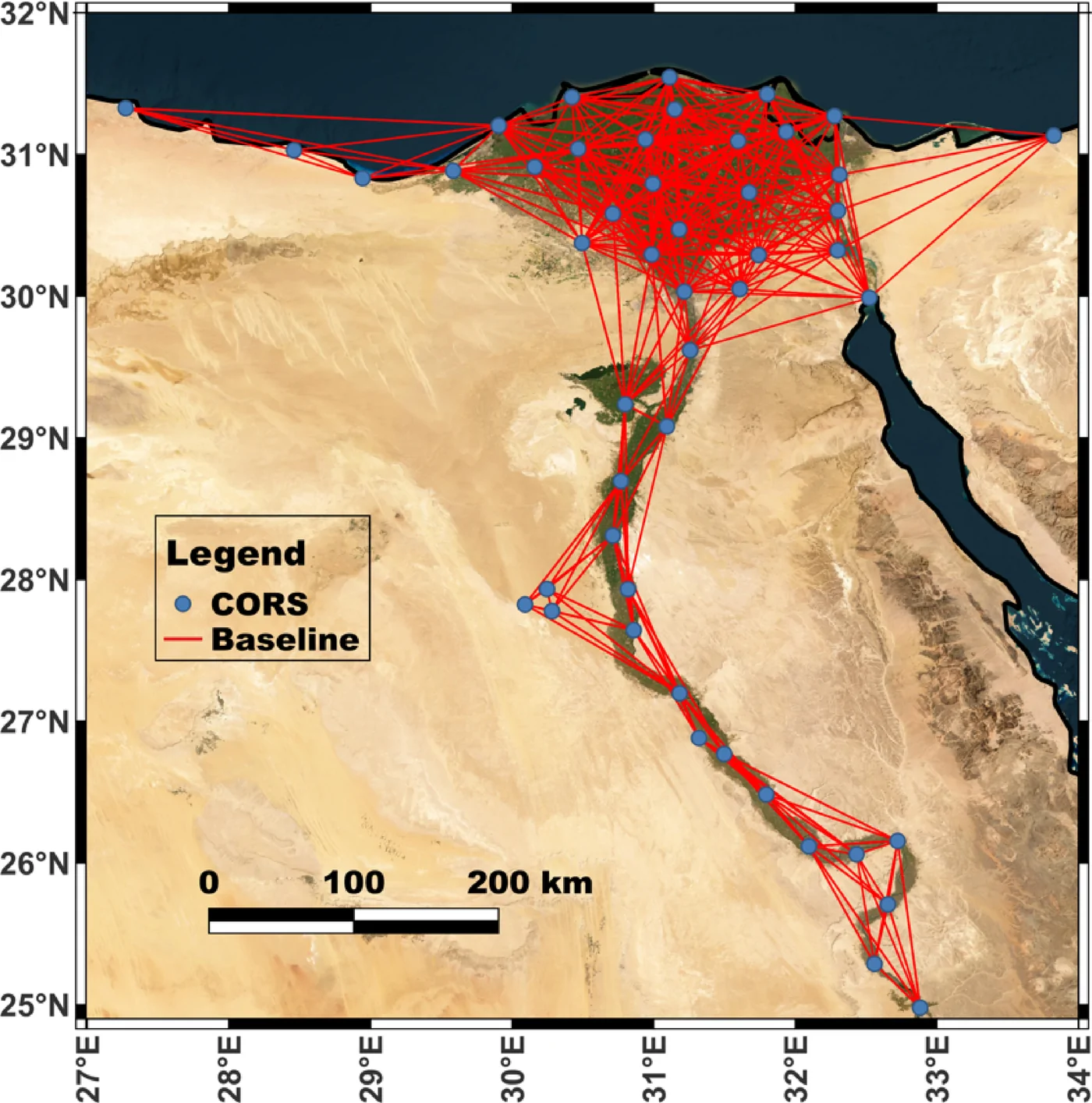
The processed 747 baselines in the Egyptian CORS network. *Generated using QGIS version 3.42.2 *(https://qgis.org).

To derive the required elements in * A* and * L*, 747 baselines in the Egyptian CORS network were processed using GNSS observations collected on different days between May and August 2025, see Fig. 4. The 747 processed baselines do not represent all possible station combinations. Instead, a baseline selection strategy was used to ensure geodetic representation while maintaining reliable baseline processing. In general, baselines longer than 150 km were excluded from the analysis to avoid the increased uncertainties associated with very long baselines and to better represent the spacings expected in the proposed network extensions. For each station, all baselines connecting with neighbouring stations within 150 km were selected. If fewer than five baselines satisfied this criterion, the five nearest stations were included to ensure sufficient redundancy.

All baselines were processed individually in double-difference relative mode^19^, using the Net_Diff (version 1.17) scientific software package^31,32^. The RTK processing mode of Net_Diff was used for all baseline processing sessions, employing Differential Code Bias (DCB) corrections, antenna phase center offsets and variations, and precise orbit products^16^. Statistics of the derived parameters from the baseline processing are presented in Table 3.

**Table 3:** Statistics of the derived parameters from the processing of 747 GNSS baselines in the Egyptian CORS network.

Parameter	$\:{\sigma\:}_{b}$ (cm)	$\:{l}_{b}$ (km)	* T* (hr)	* SV*	* PDOP*
Min.	0.5	17.62	0.68	7	1.72
Max.	5.3	251.24	20.24	23	11.12
Average	1.6	92.71	12.24	12.3	3.1

As presented in Table 3, the variability in the values of $\:{l}_{b}$, * T*, * SV*, and * PDOP* is adequate for least-squares estimation. This diversity is beneficial, as it yields a well-conditioned dataset that supports stable parameter estimation under varying observational and geometric conditions. Despite the wide data ranges, the average $\:{\sigma\:}_{b}$ value was 1.6 cm, indicating strong measurement quality and ensuring the robustness and reliability of the derived least-squares solution. By incorporating the $\:{\sigma\:}_{b}$, $\:{l}_{b}$, * T*, * SV*, and * PDOP* values of the 747 baselines into Eq. (3), the required coefficients are obtained, as presented in Table 4.

**Table 4:** The required coefficients for estimating the baseline accuracies, derived from processing the data from the recent adjustment of the Egyptian CORS network.

Coefficient	$\:{\alpha\:}_{o}$	$\:{\alpha\:}_{1}$	$\:{\alpha\:}_{2}$	$\:{\alpha\:}_{3}$	$\:{\alpha\:}_{4}$
Value	0.05153007	0.00000002	− 0.00641828	− 0.00192	0.000197

From the deduced coefficients, it is evident that all parameters have positive signs except for the coefficients of the observation duration and the number of satellites. These negative signs are expected, as increasing the observation time generally reduces the baseline’s standard errors, and the same applies to increasing the number of observed satellites.

To estimate the positional errors of the baselines in this study, representative operating conditions were adopted based on the statistical analysis of the processed GNSS observations. The adopted number of satellites and PDOP correspond to the average values derived from the existing Egyptian CORS network, see Table 3. Although the average observation duration was about 12 h, a duration of 4 h was intentionally adopted to provide a conservative estimate of the expected baseline accuracies. Therefore, Eq. (1) can be simplified as follows:

4 $$\:{\sigma\:}_{b}=0.0200\:+0..00000002*{l}_{b}$$

Thus, for instance, for a baseline length of 50 km, the resulting $\:{\sigma\:}_{b}$ value becomes 2.1 cm.

Finally, the standard error of any baseline can be written in terms of its three Cartesian components ($\:{\sigma\:}_{x}$, $\:{\sigma\:}_{y}$, and $\:{\sigma\:}_{z}$), such that:

5 $$\:{\sigma\:}_{b}^{2}=\:{\sigma\:}_{x}^{2}+\:{\sigma\:}_{y}^{2}+{\sigma\:}_{z}^{2}$$

The values $\:{\sigma\:}_{x}$, $\:{\sigma\:}_{y}$, and $\:{\sigma\:}_{z}$are determined based on the baseline components that can be expressed as:

6 $$\:\left[\begin{array}{c}{\varDelta\:x}_{12}\\\:{\varDelta\:y}_{12}\\\:{\varDelta\:z}_{12}\end{array}\right]=\left[\begin{array}{c}{x}_{2}\\\:{y}_{2}\\\:{z}_{2}\end{array}\right]-\left[\begin{array}{c}{x}_{1}\\\:{y}_{1}\\\:{z}_{1}\end{array}\right]$$

where $$\:{\left[\begin{array}{ccc}{x}_{1}&\:{y}_{1}&\:{z}_{1}\end{array}\right]}^{T}$$ and $$\:{\left[\begin{array}{ccc}{x}_{2}&\:{y}_{2}&\:{z}_{2}\end{array}\right]}^{T}$$ represent the coordinates of the baseline ends.

To estimate the precision of the computed coordinates in the proposed networks, Eq. (6) is expanded to include all points in each network. For * m* observed baselines, the Variance-Covariance (VC) matrix of the observations ($\:{{\Sigma\:}}_{L}$) can be formulated as:

7 $$\:{{\Sigma\:}}_{L}=diag({\sigma\:}_{{x}_{1}}^{2},\:{\sigma\:}_{{y}_{1}}^{2},\:{\sigma\:}_{{z}_{1}}^{2},\:{\sigma\:}_{{x}_{2}}^{2},\:{\sigma\:}_{{y}_{2}}^{2},\:{\sigma\:}_{{z}_{2}}^{2},\dots\:,\:{\sigma\:}_{{x}_{m}}^{2},\:{\sigma\:}_{{y}_{m}}^{2},\:{\sigma\:}_{{z}_{m}}^{2})$$

where $\:{\sigma\:}_{{x}_{m}}^{2}$, $\:{\sigma\:}_{{y}_{m}}^{2}$, and $\:{\sigma\:}_{{z}_{m}}^{2}$ are the variances of baseline * m* in the three Cartesian directions. The corresponding weight matrix (* P)* can be expressed as:

8 $$\:P={\sigma\:}_{o\:\:}^{2}{{\Sigma\:}}_{L}$$

in which the variance factor $\:{\sigma\:}_{o\:\:}^{2}$is assumed unity. The coefficient matrix for this linear mathematical model combines zeros and ± ones (i.e., zeros for the constrained stations in the network, while ± ones for the new stations).

The VC matrix of the unknowns ($\:{\Sigma\:}\widehat{x}$) is computed as^26^:

9 $$\:{\Sigma\:}\widehat{x}={\left({A}^{T}{P}^{-1}A\right)}^{-1}$$

The computed VC matrix can be transformed from the Cartesian frame to the local topocentric frame (East, North, and up) as follows^18^:

10 $$\:{{\Sigma\:}}_{ENU}=\left[\begin{array}{ccc}{\sigma\:}_{E}^{2}&\:{\sigma\:}_{EN}&\:{\sigma\:}_{EU}\\\:{\sigma\:}_{NE}&\:{\sigma\:}_{N}^{2}&\:{\sigma\:}_{NU}\\\:{\sigma\:}_{UE}&\:{\sigma\:}_{UN}&\:{\sigma\:}_{U}^{2}\end{array}\right]=\:{R}_{ENU}\:{\Sigma\:}\widehat{x}\:{R}_{ENU\:}^{T}$$

where $\:{{\Sigma\:}}_{ENU}$ represents the VC matrix of the unknowns in the local frame. $\:{\sigma\:}_{E}$, $\:{\sigma\:}_{N}$, and $\:{\sigma\:}_{U}$ are the standard errors in the East, North, and up directions, respectively. $\:{\mathrm{R}}_{ENU}$ is the utilized rotation matrix that can be computed as^22^:

11 $$\:{R}_{ENU}=\:\left[\begin{array}{ccc}-\mathrm{sin}\lambda\:&\:\mathrm{cos}\lambda\:&\:0\\\:-\mathrm{sin}\varphi\:\mathrm{cos}\lambda\:&\:-\mathrm{sin}\varphi\:\mathrm{sin}\lambda\:&\:\mathrm{cos}\varphi\:\\\:\mathrm{cos}\varphi\:\mathrm{cos}\lambda\:&\:\mathrm{cos}\varphi\:\mathrm{sin}\lambda\:&\:\mathrm{sin}\varphi\:\end{array}\right]$$

where $\:\varphi\:$ and $\:\lambda\:$ denote the point latitude and longitude. Finally, the positional standard error ($\:{\sigma\:}_{P}$) of any proposed station is computed as:

12 $$\:{\sigma\:}_{P}=\sqrt{{\sigma\:}_{E}^{2}+{\sigma\:}_{N}^{2}+{\sigma\:}_{U}^{2}}$$

## The estimated positional standard errors of the proposed CORS stations

This section presents the results obtained after applying the strategy described in Sect. 3 to estimate the positional standard errors of the proposed CORS stations. The standard errors ($\:{\sigma\:}_{E}$, $\:{\sigma\:}_{N}$, $\:{\sigma\:}_{U}$, and $\:{\sigma\:}_{P}$) are calculated for the proposed stations in the five zones.

**Fig. 5:**
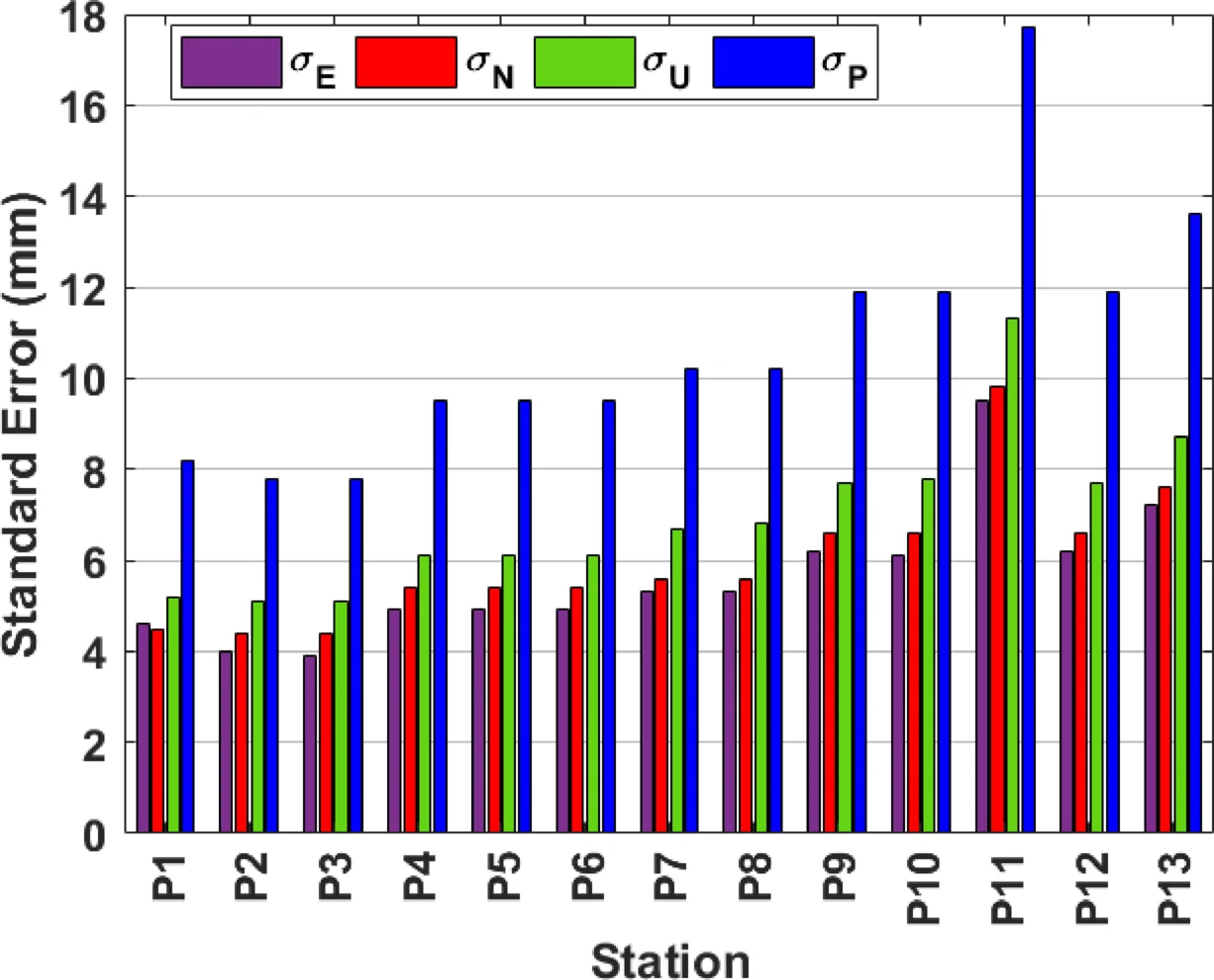
The computed standard errors of the proposed CORS stations in the New Delta zone.

In the New Delta zone, Eq. (4)–(10) are applied for stations P1–P13. The computed standard errors are depicted in Fig. 5, while Table 5 summarizes the statistics of these errors.

**Table 5:** Statistics of the positional standard errors of the proposed stations in the New Delta zone.

Value	$\:{\boldsymbol{\sigma\:}}_{\boldsymbol{E}}$ (mm)	$\:{\boldsymbol{\sigma\:}}_{\boldsymbol{N}}$ (mm)	$\:{\boldsymbol{\sigma\:}}_{\boldsymbol{U}}$ (mm)	$\:{\boldsymbol{\sigma\:}}_{\boldsymbol{P}}$ (mm)
Minimum	3.9	4.4	5.1	7.8
Maximum	9.5	9.8	11.3	17.7
Average	5.6	6.0	6.9	10.8
Standard deviation	1.5	1.5	1.7	2.7

From Fig. 5; Table 5, it is clear that all standard errors do not exceed 12 mm in the three positional components, leading to three-dimensional positional errors of less than 18 mm. The average positional errors in the east, north, and up directions show promising behavior, with values of 5.6 mm, 6.0 mm, and 6.9 mm, respectively. Moreover, Fig. 5 shows that the terminal stations farthest from the existing CORS (i.e., P9, P10, P11, P12, and P13) exhibit the largest standard error values, which is expected.

**Fig. 6:**
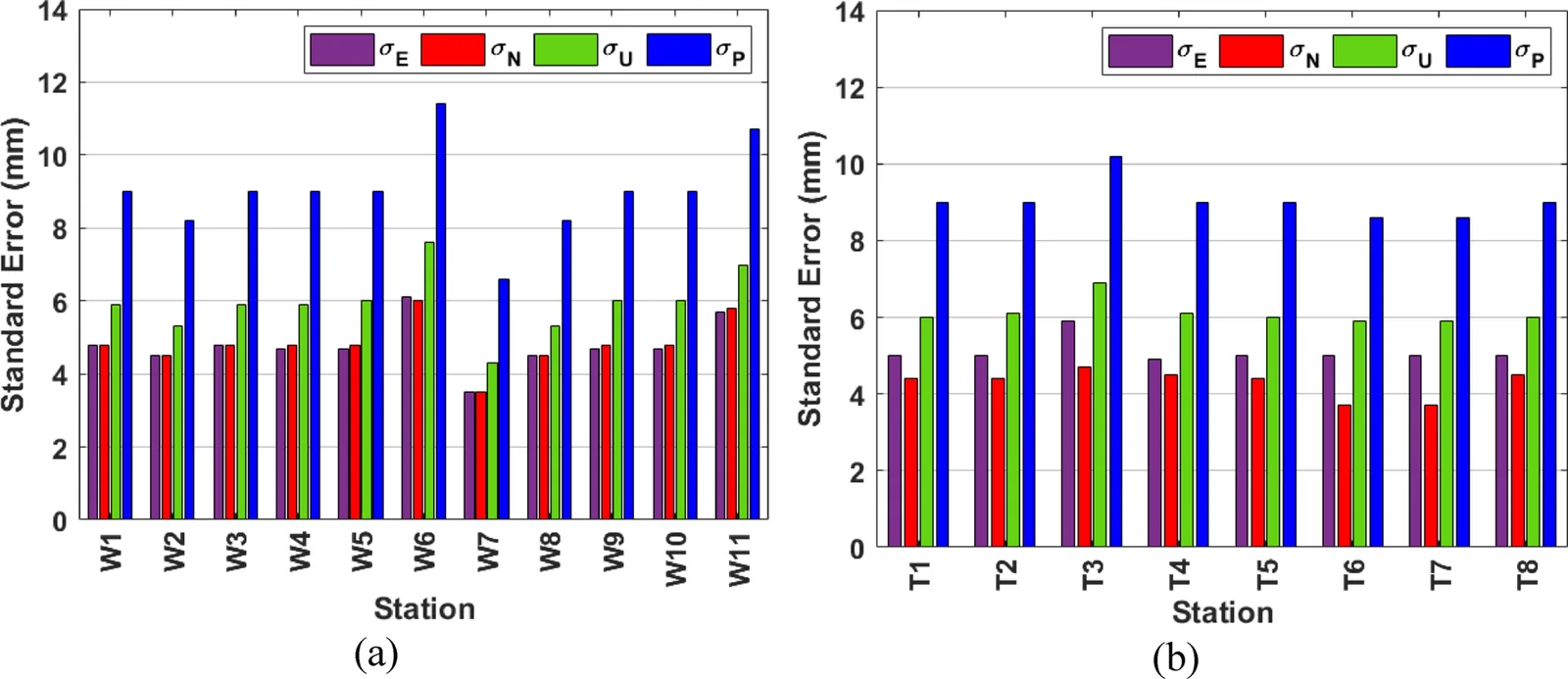
The computed standard errors of the proposed CORS stations in (**a**) the western zone of the North Coast (**b**) Toshka.

The same computational strategy is applied to the proposed stations in the western zone of the North Coast and in Toshka. Figure 6 presents the detailed standard errors of each station in both zones. In addition, Tables 6 and 7 give the statistics of the resulting positional standard errors in the western zone of the North Coast and Toshka, respectively.

**Table 6:** Statistics of the positional standard errors of the proposed stations in the western zone of the North Coast.

Value	$\:{\boldsymbol{\sigma\:}}_{\boldsymbol{E}}$ (mm)	$\:{\boldsymbol{\sigma\:}}_{\boldsymbol{N}}$ (mm)	$\:{\boldsymbol{\sigma\:}}_{\boldsymbol{U}}$ (mm)	$\:{\boldsymbol{\sigma\:}}_{\boldsymbol{P}}$ (mm)
Minimum	3.5	3.5	4.3	6.6
Maximum	6.1	6.0	7.6	11.4
Average	4.8	4.8	5.9	9.0
Standard deviation	0.7	0.7	0.9	1.3

**Table 7:** Statistics of the positional standard errors of the proposed stations in Toshka.

Value	$\:{\boldsymbol{\sigma\:}}_{\boldsymbol{E}}$ (mm)	$\:{\boldsymbol{\sigma\:}}_{\boldsymbol{N}}$ (mm)	$\:{\boldsymbol{\sigma\:}}_{\boldsymbol{U}}$ (mm)	$\:{\boldsymbol{\sigma\:}}_{\boldsymbol{P}}$ (mm)
Minimum	4.9	3.7	5.9	8.6
Maximum	5.9	4.7	6.9	10.2
Average	5.1	4.3	6.1	9.1
Standard deviation	0.3	0.4	0.3	0.5

From Fig. 6a, it can be noticed that points W6 and W11 exhibit the largest standard errors, as they represent the terminal stations on both sides. The remaining stations achieve lower standard error values due to the constrained conditions imposed by the MTRH, OZ21, and OZ22 stations (see Fig. 3b). In addition, the statistics in Table 6 show that the three-dimensional positional standard error for all stations remains below the 12 mm level. The average three-dimensional standard error across all stations is 9 mm.

In the Toshka network, Fig. 6b and Table 7 indicate only slight variations in the standard errors over all stations. The difference between the minimum and maximum standard error values is 1 mm in the three directions. The average standard errors are 5.1 mm, 4.3 mm, and 6.1 mm in the east, north, and up directions, respectively. Moreover, the maximum three-dimensional positional standard error is 10.2 mm, while the average value is 9.1 mm.

**Fig. 7:**
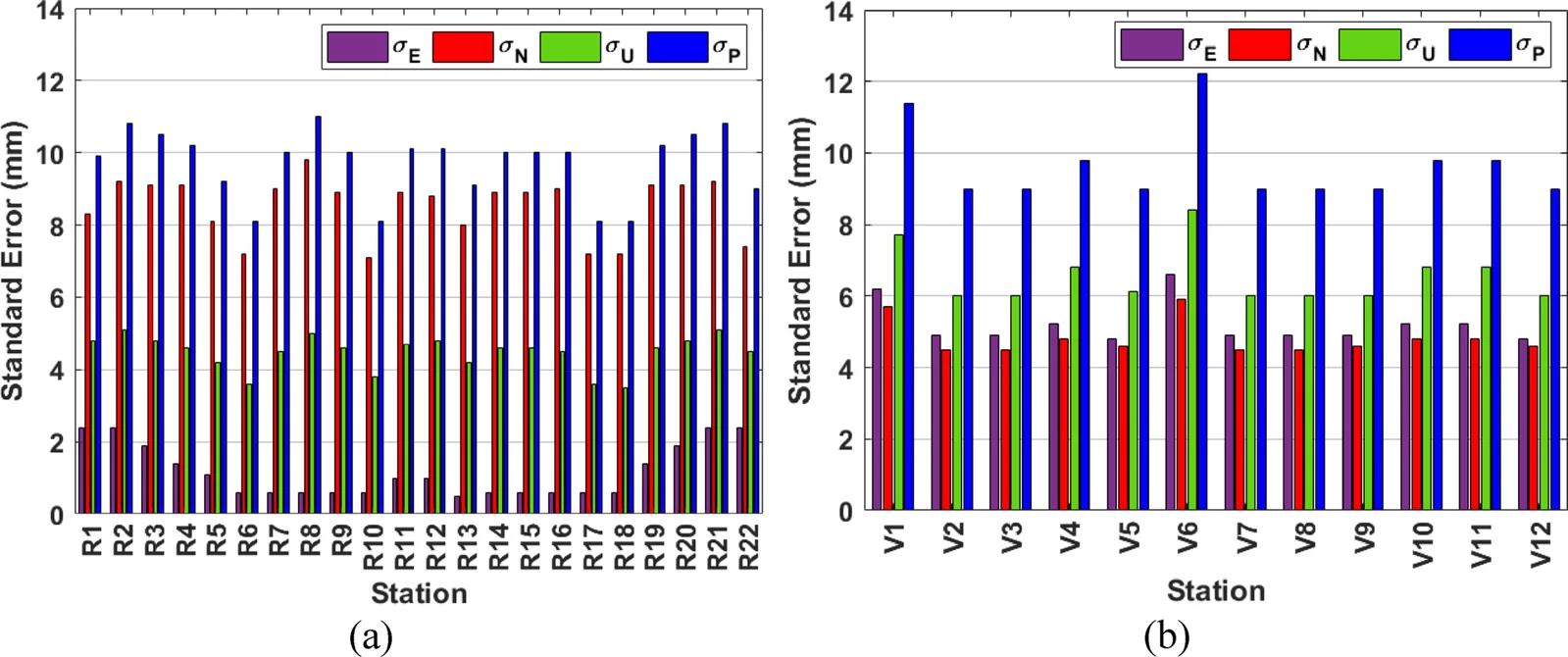
The computed standard errors of the proposed CORS stations (**a**) on the Red Sea Coast (**b**) in the New Valley zone.

The same analysis is subsequently applied to the proposed stations on the Red Sea Coast and in the New Valley zone. Figure 7 presents a detailed assessment of the standard errors for each station in both zones, whereas Tables 8 and 9 report the statistical characteristics of the resulting positional standard errors for the Red Sea Coast and New Valley zones, respectively.

**Table 8:** Statistics of the positional standard errors of the proposed stations on the Red Sea Coast.

Value	$\:{\boldsymbol{\sigma\:}}_{\boldsymbol{E}}$ (mm)	$\:{\boldsymbol{\sigma\:}}_{\boldsymbol{N}}$ (mm)	$\:{\boldsymbol{\sigma\:}}_{\boldsymbol{U}}$ (mm)	$\:{\boldsymbol{\sigma\:}}_{\boldsymbol{P}}$ (mm)
Minimum	0.5	7.1	3.5	8.1
Maximum	2.4	9.8	5.1	11.0
Average	1.2	8.5	4.5	9.7
Standard deviation	0.7	0.8	0.5	1.0

**Table 9:** Statistics of the positional standard errors of the proposed stations in the New Valley zone.

Value	$\:{\boldsymbol{\sigma\:}}_{\boldsymbol{E}}$ (mm)	$\:{\boldsymbol{\sigma\:}}_{\boldsymbol{N}}$ (mm)	$\:{\boldsymbol{\sigma\:}}_{\boldsymbol{U}}$ (mm)	$\:{\boldsymbol{\sigma\:}}_{\boldsymbol{P}}$ (mm)
Minimum	4.8	4.5	6.0	9.0
Maximum	6.6	5.9	8.4	12.2
Average	5.2	4.8	6.6	9.7
Standard deviation	0.6	0.5	0.8	1.1

Figure 7a and Table 8 show minor station-to-station standard error variations, indicating that the Red Sea Coast network is well-conditioned. As the network extends predominantly in the north-south direction, the average standard error in the east direction is only 1.2 mm, while the corresponding values in the north and up directions are 8.5 mm and 4.5 mm, respectively. Although the network extends over more than 500 km, constraining it using three stations (0Z08, 0Z09, and 0Z14) maintains the positional standard error below 1.1 cm.

In the New Valley zone, the results in Fig. 7b indicate that the standard error behavior is almost uniform, reflecting a stable adjustment solution. Stations V1 and V6 exhibit slightly larger standard error values, as they represent terminal stations constrained from only one side. The statistics in Table 9 show that the positional standard error is kept below 12.2 mm, with an average value of 9.7 mm.

It should be noted that the proposed network extensions were assessed under the existing operational parameters. Therefore, the results and analysis presented in this study are based on the current 49 stations of the Egyptian CORS network, operating with GPS and GLONASS observations. As the network evolves and additional GNSS constellations (i.e., Galileo and BeiDou) are incorporated through future station modernization, further processing and analysis will be required to quantify the improvements in positioning accuracy afforded by multi-constellation observations.

Overall, the results presented in this study demonstrate the robustness and consistency across the proposed extensions to the Egyptian CORS network. Despite the different extension sizes and constraint configurations, the positional standard errors remain below 1.8 cm, emphasizing the effectiveness of the applied constraints. The indicated stable error behavior confirms the suitability of the proposed network extensions for centimeter-level positioning applications in the targeted regions, in support of Egypt’s Vision 2030.

The proposed framework in this study follows the same general strategy adopted by national CORS networks in other countries. Similar to the National Oceanic and Atmospheric Administration (NOAA) CORS Network in the United States^11^ and the SAPOS network in Germany^24^, the proposed Egyptian network extensions aim to provide nationwide coverage through strategically distributed stations. Likewise, the proposed extensions prioritize improved spatial coverage of developing regions while maintaining the geodetic integrity of the national reference datum. Such extensions are expected to provide several practical benefits for end users by improving the availability and reliability of high-accuracy GNSS positioning for surveying and mapping, infrastructure development, engineering and construction, precision agriculture, environmental monitoring, and scientific research.

A limitation of the present results is that the proposed extensions were assessed using mathematical modeling and baseline accuracy estimates rather than field observations at the proposed station locations, as these stations have not been established yet. Consequently, experimental validation of the predicted network performance is not currently feasible. Similar CORS network design studies (e.g.,^2,14^ likewise relied on pre-deployment network planning and analytical evaluation, conducting field validation only after network establishment. Following the implementation of the proposed network extensions, future work should include comprehensive experimental validation using real GNSS observations to evaluate positioning performance and verify predicted accuracies under operational conditions.

Although the main objective of this study is the geodetic design and evaluation of the proposed CORS network extensions, a preliminary economic assessment is important to indicate the required investment. The implementation of a permanent GNSS station includes several elements, mainly the monument construction, GNSS receiver and antenna, power supply, communications infrastructure, installation, and commissioning. Published guidelines indicate that the establishment cost of a single CORS is typically on the order of 25,000–35,000 USD, depending on equipment specifications and site conditions (UNAVCO,^29^. Based on an average estimate, the proposed addition of 66 new CORS would require an initial capital investment of about 2 million USD. Recent studies estimate the annual operation and maintenance costs to be about 10% of the initial investment per year^17^. Despite this investment, the proposed network extensions are expected to provide substantial long-term economic benefits by supporting the national infrastructure and development projects in the targeted regions, as discussed in Sect. 1. The potential economic returns are expected to substantially outweigh the initial investment and recurring operational/maintenance costs.

## Conclusions

Despite recent development initiatives in Egypt, the existing CORS network remains concentrated mainly in the Nile Delta and Nile Valley regions, leaving the new development areas largely uncovered. This study proposed strategic extensions to the Egyptian CORS network, in alignment with the country’s national development vision. The targeted regions include the New Delta, the western zone of the North Coast, Toshka, the New Valley, and the Red Sea Coast.

The proposed framework builds upon the existing Egyptian CORS network, which consists of 49 stations, and recommends the addition of 66 new stations to expand the network to a total of 115 stations, thereby extending coverage to the targeted regions. For the proposed extensions, station locations were selected to form triangular and quadrilateral baseline configurations, with spacings ranging from 15 to 95 km. The expected positioning accuracies of the proposed extensions were estimated through rigorous GNSS data processing and network analysis, using the existing CORS configuration.

To assess the network accuracy, GNSS observations from the existing 49 stations were collected and processed to derive the coefficients governing baseline accuracy estimation. The results demonstrate the robustness and consistency of the proposed extensions, with positional standard errors below 1.8 cm. The stable error behavior across all planned extensions indicates their suitability for regional geodetic infrastructure and centimeter-level positioning applications in the targeted regions, supporting Egypt’s Vision 2030. A limitation of this study is the lack of experimental validation, as the proposed CORS have not been established yet. However, the framework provides a robust foundation for network planning, and future work will evaluate the predicted performance using real GNSS observations once implemented.

The proposed extensions will enhance GNSS positioning availability and reliability, directly benefiting key sectors such as surveying, infrastructure, construction, precision agriculture, environmental monitoring, and scientific research. Moreover, a preliminary economic analysis indicates that these extensions represent a justified long-term investment. The potential economic returns are expected to greatly surpass the initial investment and recurring costs.

## Data Availability

The data and codes used in this study are available from the corresponding author upon reasonable request.
